# Predicting Caregiver Burden in Informal Caregivers for the Elderly in Ecuador

**DOI:** 10.3390/ijerph17197338

**Published:** 2020-10-08

**Authors:** Pablo Ruisoto, Marina Ramírez, Belén Paladines-Costa, Silvia Vaca, Vicente Javier Clemente-Suárez

**Affiliations:** 1 Department of Health Sciences, University of Navarre, 31006 Pamplona, Spain; pablo.ruisoto@unavarra.es; 2 Department of Psychology, Universidad Técnica Particular de Loja, Loja 110107, Ecuador; mbpaladines@utpl.edu.ec (B.P.-C.); slvaca@utpl.edu.ec (S.V.); 3 Faculty of Sport Sciences, Universidad Europea de Madrid, 28670 Villaviciosa de Odón, Spain; vicentejavier.clemente@universidadeuropea.es; 4 Grupo de Investigación en Cultura, Educación y Sociedad, Universidad de la Costa, Barranquilla 080002, Colombia

**Keywords:** stress, burden, informal caregivers, burden, elderly, gender differences, neuroticism, competence

## Abstract

Informal caregivers are the main providers of care for the elderly. The aim of this study is to examine the predictive value of different variables regarding caregivers and their elderly patients with respect to the caregiver’s burden. A convenience sample of 688 informal caregivers and 688 elderly people from Ecuador was surveyed. Only households with one caregiver and one elderly person were considered for the study. For informal caregivers, the following standardized measures were obtained: burden (Zarit Burden Interview), neuroticism (Eysenck Personality Questionnaire Revised-Abbreviated, EPQR-A), caregiver’s general health (GHQ-12), and social support (modified Duke-UNC Functional Social Support Questionnaire, FSSQ11). For the elderly, we employed standardized measures of cognitive function (short portable mental status questionnaire, SPMSQ), Pfeiffer’s test, and functional dependency (Barthel scale/Index, BI). Females were over-represented in caregiving and reported significantly higher burden levels than those of males. In both male and female caregivers, the burden was best predicted by the time of caring, neuroticism, and elderly cognitive impairment. However, some predictors of burden were weighted differently in males and females. The functional independence of the elderly was a significant predictor of burden for male caregivers but not females, while caregiver competence was a significant predictor for females but not males. These variables accounted for more than 88% of the variability in informal caregivers.

## 1. Introduction

Stress is an intense and unusual stimulus elicited by the presence of a threat, or any other circumstance or event that an individual perceives as adverse [1]. There are some professions that elicit a large stress response, modifying the homeostasis of the natural organism. In professions such as the military, police, firefighting, and even among elite athletes, individuals frequently experience stress responses beyond the body’s natural limits [2,3,4]. However, stress is not only present in these professions; medical personnel, drivers, journalists, and even teachers and students exhibit elevated stress levels as a result of their exposure to their work context [5,6,7].

As an acute response, stress is designed to maintain the physical integrity of the subject; however, continuous exposure to stress may result in psychopathologies such as anxiety, depression, post-traumatic stress disorder and burnout [8].

In this respect, stress-related health issues as a result of the burden of providing care are well-documented in informal or family caregivers [9,10,11,12,13]. Moreover, the risk in the informal caregivers of the elderly is increasing since the general population age is steadily increasing. Providing care to the elderly is an important source of chronic stress considering the demands of providing care to elderly patients and the lack of resources of informal caregivers, including formal training.

In the context of caregiving, the stress model underlines the importance of the perceived lack of control and the psychological stress involved in the situation of caring compared with the objective amount of expected burden as a result of the degree of mental impairment or dependency of the elderly patient [14]. Some authors suggested that the time spent providing care, the caregiver’s age, their lack of social support, the cognitive impairment of the elderly, and the caregiver’s neuroticism are important factors [15,16]. Previous studies also found differences in the amount of perceived burden in caregivers [17]. However, most studies focused on caregivers of people with dementia [18,19,20] or other mental disorders [21].

In sum, the best predictors of burden in informal caregivers of the elderly in Ecuador remain understudied and elusive. The objective of this study is to analyze the predictive value of variables regarding caregivers and their elderly patients with respect to the burden of informal caregivers of the elderly. Furthermore, gender differences were analyzed. To our knowledge, this is the largest study attempting to predict burden in informal caregivers in Ecuador. This research could contribute to the development of interventions aimed at improving the well-being of caregivers of the elderly.

## 2. Materials and Methods

### 2.1. Participants

A convenience sample of 688 family caregivers (mean age = 49.1 ± 14.6; 79.8% females) and their respective 688 elderly patients (mean age = 80.8 ± 9.2; 60.5% females) from eight regions of Ecuador were recruited for this study. All participants were selected from the user referral lists of local retirement associations and centers associated with the Ministry of Social and Economic Inclusion (MIES). As inclusion criteria, all caregivers were relatives of their elderly patients, and their role was that of primary caregiver without receiving any compensation for this service, taking on responsibility for any decisions and for their patient’s well-being for at least 12 months. None of the caregivers was receiving specialized psychological support at the time of assessment, and they were living at home with the elderly people. All subjects had given their informed consent for inclusion before they participated in the study. The study was conducted in accordance with the Declaration of Helsinki and was approved by the local committee at the Public University of Loja (code 05-04/02/2019).

### 2.2. Measures

All caregivers were interviewed in one session divided into two parts: a sociodemographic questionnaire (age, gender, time providing care, marital status, ethnic, education level), and a psychological protocol using instruments developed and/or validated in Spanish. Specifically, the following standardized measures were included in this survey:

The Zarit Burden Interview [22] consists of 22 items measuring burden in caregivers. Participants respond using a Likert scale, ranging from 1, “never” to 5, “always”. Scores range from 22 to 110. One example item is “I think people I am providing care to are asking for more help than they need”. The internal consistency for this study was high, with a Cronbach’s alpha of 0.92.

CUIDAR [23] consists of 20 items assessing the caregiver’s competency to provide care. Participants respond using a Likert scale ranging from 0, “never” to 3, “always or almost always”. Scores range from 0 to 60. An example item is “I know how to monitorize the health condition of the person to whom I provide care”. The internal consistency for this study was high, with a Cronbach’s alpha of 0.88.

The Spanish Version of the Brief COPE (COPE-28) [24] consists of 28 items and assesses different coping scales, including substance use and religion. Participants respond using a four-point scale ranging from 0, “I have not done that at all” to 3, “I have done that very often”. Scores ranged from 0 to 6 for each subscale. An example item related to substance use is “I have taken alcohol or other drugs to feel better”, and an example related to religion is “I have tried to find comfort in my religion or spiritual beliefs”. The internal consistency for this study was high, with a Cronbach’s alpha of 0.83 (substance use α = 0.62; religion α = 0.75).

Duke-UNC Functional Social Support Questionnaire (FSSQ11) [25,26] consists of 11 items aiming to assess perceived social support. Participants respond using a scale ranging from 0, “never”, to 5, “always”. Scores range from 0 to 55. An example item is “I receive visits from friends and family”. The internal consistency for this study was high, with a Cronbach’s alpha of 0.91.

Personality Questionnaire Revised-Abbreviated (EPQR-A) [27] consists of 24 items aiming to assess personality traits, including neuroticism, one of the big five higher-order personality traits. Scores range from 0 to 6 for each scale. An example item is “I suffer significant changes in my mood”. Internal consistency for this study was good, with a Cronbach’s alpha ranging from 0.63 to 0.78.

The Barthel scale/index (BI) [28] consists of 10 items assessing functional dependency in daily life activities. Participants answer using a scale ranging from 0), dependent, to (5), need help, to (10), independent. Scores range from 0 to 100. An example item is “Help is needed to eat”. The internal consistency for this study was high, with a Cronbach’s alpha of 0.93.

Short portable mental status questionnaire (SPMSQ) [29,30] consists of 10 items aiming to assess cognitive impairment. Scores range from 0 to 10. An example item is “What day is today? (month, day, and year)”. The internal consistency for this study was high, with a Cronbach’s alpha of 0.89.

General Health Questionnaire (GHQ-12) [31] consists of 12 items, each assessing the severity of a mental problem over the past few weeks using a four-point Likert scale (from 0 to 3). An example item is “I have recently felt that I am ill”. The score was used to generate a total score ranging from 0 to 36. The internal consistency for this study was high, with a Cronbach’s alpha of 0.94.

### 2.3. Design and Procedure

**Figure 1:**
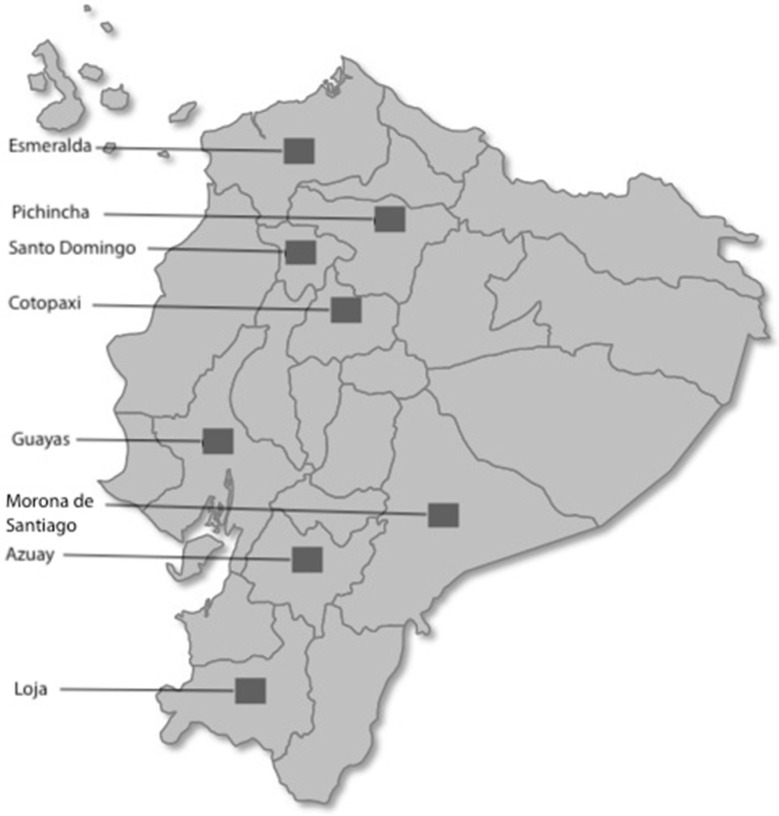
Distribution of the data-collection areas in Ecuador.

A cross-sectional correlational study was conducted. Data were collected from eight regions in Ecuador in late 2019. For spatial distribution of the areas in Ecuador from which data were collected, see Figure 1. All data were collected via a survey that included standardized sociodemographic scales, which was administered using printed material by a team of psychologists trained by the lead researcher. Data from caregivers and the elderly were collected separately (first from the caregiver, then from the elderly). The duration of the sessions averaged 20–25 min.

### 2.4. Data Analysis

Statistical analyses were performed using the Statistical Package for Social Sciences application, version 21.0 for Mac (IBM, Madrid, Spain). The sociodemographic and clinical characteristics of the groups were expressed as means (M) and standard deviations (SD). Pearson’s correlation was conducted to examine the relationship between measured informal caregiver burden, and variables pertaining to the caregivers and their elderly patients. Inclusion in the prediction models of burden in informal male and female caregivers (enter method) was based on correlation analysis between burden and target variables. Student’s t-test (independent samples) was used to determine significant gender differences for quantitative variables. Levene’s and Shapiro–Wilk’s tests were used to assess the homogeneity of variance and normality, respectively. Effect size was measured using Cohen’s d. Lastly, independent multiple-regression models were conducted, including measured variables pertaining to caregivers and their elderly patients (predictive variables) and burden as the outcome variables. Significance adopted in analysis was *p* < 0.05.

## 3. Results

### 3.1. Description of Caregiver and Elderly Samples

The final sample was made up of 688 family caregivers and 688 elderly patients from eight regions of Ecuador: 7.7% from Guayas (*n* = 53), 7.7% from Morona Santiago (*n* = 53), 2.9% from Esmeraldas (*n* = 20), 11.9% from Loja (*n* = 82), 8.7% from Azuay (*n* = 60), 4.5% from Cotopaxi (*n* = 31), 7.7% from Santo Domingo (*n* = 53), and 48.9% from Pichincha (*n* = 336), the closest area to the capital of the country, Quito. The caregivers’ relationship to their elderly patients was as follows: 12.5% (*n* = 86) were husband/wife, 63.8% (*n* = 439) were son/daughter, 13.4% (*n* = 92) were grandson/granddaughter, 5.5% (*n* = 38) were brother/sister, and 4.8% (*n* = 33) were daughter/son-in-law. Caregiver age ranged from 17 to 80 years old, and the age of elderly patients (older than 65 years old) ranged from 65 to 104 years old.

Females were over-represented among family caregivers. Both male and female caregivers provided care for the elderly for an average of 6 years. Considering their age at the time of the study, this means that most caregivers of the elderly in Ecuador assume their role in their early 40 s, regardless of their educational level (Table 1).

### 3.2. Gender Differences in Caregiver and Elderly Variables Associated with Caregiver Burden

Female caregivers reported higher statistically significantly degrees of burden and neuroticism than those of male caregivers. Females mostly cope through religion, and males through substance abuse (Table 2).

### 3.3. Pearson’s Correlation between Informal Caregivers’ Burden and Measured Variables

For informal male caregivers of the elderly, the degree of burden in informal caregivers was positively correlated with time providing care (*r* = 0.216, *p* = 0.012) and neuroticism (*r* = 0.316, *p* < 0.001), and negatively correlated with caregiver’s social support (*r* = −0.365, *p* < 0.001) and competence as caregiver (*r* = −0.488, *p* > 0.001). However, burden in informal male caregivers was not significantly associated with the perception of mental-health problems, as measured by the GHQ-12 (*r* = 0.026, *p* = 0.768), which, in fact, was positively and significantly associated with neuroticism (*r* = 0.319, *p* < 0.001). Interestingly, neither functional dependency (*r* = −0.0688, *p* = 0.104) nor cognitive impairment (*r* = 0.104, *p* = 0.0234) were significantly associated with burden, although they were significantly associated with each other (*r* = 0.480, *p* < 0.001).

For informal female caregivers of the elderly, the degree of burden in informal caregivers was positively correlated with time providing care (*r* = 0.146, *p* < 0.001), neuroticism (*r* = 0.367, *p* < 0.001), functional dependency (*r* = –0.173, *p* > 0.001), and cognitive impairment (*r* = 0.181, *p* < 0.001), and negatively correlated with caregiver’s social support (*r* = −0.302, *p* < 0.001) and competence as caregiver (*r* = −0.348, *p* > 0.001). However, burden in informal female caregivers was not significantly associated with the perception of mental-health problems, as measured by the GHQ-12 (*r* = 0.027, *p* = 0.537), which, in fact, was positively and significantly associated with neuroticism (*r* = 0.387, *p* < 0.001).

### 3.4. Prediction Models of Burden in Informal Caregivers of the Elderly

For informal male caregivers of the elderly, regression-model data indicated that the best predictors of burden were time providing care, the caregiver’s neuroticism, and the degree of the elderly’s functional dependency and cognitive impairment. The model accounted for 87.9% of the variance in burden (F_Δ8114_ = 457.367, *p* > 0.001). Competence as caregiver and social support failed to significantly predict burden in this model (Table 3).

For informal female caregivers, regression-model data indicated that the best predictors of burden were time providing care, caregiver’s competence, caregiver’s neuroticism, and elderly’s cognitive impairment. The model accounted for 88.1% of the variance in burden (F_Δ8496_ = 92.816, *p* > 0.001) (Table 4).

## 4. Discussion

The aim of this study was to examine the predictive value of variables pertaining to caregivers and their elderly patients with respect to caregiver burden. To our knowledge, this is the largest study attempting to predict burden in informal caregivers in Ecuador and contributes to the development of interventions aimed at improving the well-being of caregivers of the elderly.

The results of this study suggest that some gender differences may be relevant in the prediction of burden in informal caregivers of the elderly. For males, objective strains that are directly related with providing care seem to be more relevant, for example, time providing care (duration) and degree of elderly demands (both functional and cognitive) [32,33]. For females, subjective strains, in particular those related with their competence as caregivers, seem to play a more central role in the prediction of burden in this sample. This result is consistent with previous studies focusing on caregiver competence [34]. In both cases, informal male and female caregivers’ time providing care and neuroticism remained significant predictors of burden. Indeed, as expected, the duration of the stressor providing care is the most important predictor of burden in informal caregivers. Furthermore, those who were more likely to be moody and to experience feelings such as anxiety, worry, fear, anger, frustration, guilt, depressed mood, and loneliness were more likely to report higher levels of burden. This result is consistent with the previous literature [35,36].

The results of this study indicate that informal female caregivers not only double the percentage of informal caregivers for the elderly, but also report significantly higher levels of burden. This result is consistent with previous research that strongly associated females with the role of care provider [37]. These results are also in line with the previous literature where females presented greater levels of burnout syndrome than those of males in their working environment [38]. Other studies also found higher burnout, perceived stress, and emotional exhaustion in female than in male professors in colleges and high schools [39,40]. Along the same lines, some authors also found cultural differences, since the gender gap regarding emotional exhaustion is greater in females than in males, with this trend being significantly higher in female employees from the United States when compared to that in Europeans [41].

Informal male and female caregivers also differ in their main coping mechanisms [42]. Males tend to use more active stress-management mechanisms, such as those based on substance use, while females tend to rely on more passive mechanisms based on praying and hoping for the best. This result complements previous studies focusing on a higher preference for substance use in males [43]. The absence of effective coping systems in the face of caregivers’ highly demanding context on a professional and especially on an emotional level produces nonadaptive responses that can lead to drug abuse or be the basis for other psychological responses based on despair that can lead to psychopathologies such as anxiety and depression [44]. Therefore, it is important to emphasize the role of education in this professional group, not only to offer quality care, but also to preserve themselves from suffering pathologies and antisocial behaviors in the near future.

In sum, these results have potential implications for the development of social policies or recommendations to predict and reduce the burden in caregivers of the elderly or to design gender-specific interventions. This is important because providing care often results in poor health and quality of life [45,46], while at the same time, these consequences have been understudied or overlooked, so caregivers currently remain invisible patients [47]. In general, the results of this study support the importance of monitoring caregivers’ period of time providing care (i.e., their exposure to stress), for example, by including resting periods to recover, and the deleterious effects of caregivers’ neuroticism, underling the importance of moving towards a more mindful approach. Results also highlight the importance of enhancing elderly people’s cognitive function through performing cognitively stimulating tasks (e.g., chess, crosswords), and being socially and physically active. Particularly in female caregivers of the elderly, enhancing their perception of competence as caregivers seems to be an additional element to consider in reducing their burden. However, the results of this study must be taken with caution since the study is based on cross-sectional correlational design on a convenience sample; therefore, there are limitations in terms of generalization to other populations and inference of causality, and more research in this area is needed.

## 5. Conclusions

Females were over-represented with respect to males among caregivers of the elderly by a ratio of 4:1, and they reported significantly higher levels of burden than those of male caregivers. Overall, time providing care, neuroticism, and cognitive impairment of elderly patients were the best predictors of caregiver burden, and these should be considered for tailored interventions aimed at reducing burden in caregivers of the elderly.

## Figures and Tables

**Table 1:** Sociodemographic information of informal caregivers and elderly.

**Informal Caregiver Sociodemographics**	**Males** **M ± SD (*n* = 139)**	**Females** **M ± SD (*n* = 549)**
Age (years)	47.6 ± 14.8	49.6 ± 14.7
Providing care (years)	6.2 ± 4.5	5.9 ± 4.2
	% (*n*)	% (*n*)
Marital status (S/M/D/W)	41 (57)/52 (71)/11 (6.5)/0	27.1 (149)/56.6 (343)/12.6 (37)/3.6 (20)
Ethnicity (M, W, O)	82 (114)/13.7 (19)/4.3 (6)	93.4 (512)/3.5 (19)/3 (15)
Education level (B, S, C)	8.6 (12)/38.8 (54)/46 (64)	22.4 (123)/41.0 (225)/31.5 (173)
**Elderly Sociodemographics**	**Males** **M ± SD (*n* = 272)**	**Females** **M ± SD (*n* = 416)**
Age (years)	80.6 ± 9.0	81.3 ± 9.3
	% (*n*)	% (*n*)
Marital status (S/M/D/W)	5.5 (15)/60.3 (164)/0	33.7 (140)/49.5 (206)/13.7 (57)/3.1 (13)
Ethnicity (M, W, O)	89.7 (244)/7.4 (20)/3 (8)	90.6 (377)/6 (25)/3.3 (14)
Education level (B, S, C)	59.2 (161)/19.9 (54)/8.8 (24)	15.9 (66)/42.1 (175)/35.8 (149)

Marital status: S = single, M = married, D = divorced, W = widowed; ethnicity: M = mixed racial or ethnic ancestry, W = white, O = other; education level: B = basic, S = secondary, C = college.

**Table 2:** Gender differences in informal caregiver and elderly variables.

**Informal Caregiver Variables**	**Males** **(*n* = 136)** **M ± SD**	**Females** **(*n* = 541)** **M ± SD**	***t***	**df**	***p***	**Cohen’s d**
Caregiver’s burden (Zarit)	39.4 ± 17.6	45 ± 17.7	–3.300	668	0.001 **	–0.317
Caregiver’s competency (CUIDAR)	46 ± 9.7	46.5 ± 8.9	–0.455	666	0.649	–0.044
Caregiver’s general health (GHQ12)	17 ± 8.32	17.9 ± 8.9	–1.067	677	0.286	–0.102
Caregiver’s social support (DUKE11)	36.9 ± 10.4	36.4 ± 10.8	0.440	682	0.660	0.042
Caregiver’s coping style—“substance abuse” (COPE)	0.7 ± 1.4	0.3 ± 0.8	4.843	682	<0.001 **	0.460
Caregiver’s coping style—“religion” (COPE)	2.9 ± 2	3.8 ± 2	–4.609	683	<0.001 **	–0.439
Caregiver’s neuroticism (EPQRA)	2.5 ± 1.8	3.1 ± 1.8	–3.234	681	0.001 **	–0.310
Caregiver’s extraversion (EPQRA)	23.9 ± 1.6	3.7 ± 1.7	1.173	682	0.241	0.112
**Elderly Variable**	**Males** **(*n* = 266)** **M ± SD**	**Females** **(*n* = 411)** **M ± SD**	***t***	**df**	***p***	**Cohen’s d**
Elderly’s cognitive impairment (Pfeiffer)	3.6 ± 3.3	4.7 ± 3.5	−3.892	682	0.001 **	−0.304
Elderly’s functional independency (Barthel)	57.8 ± 32.71	59.3 ± 31.9	−0.601	675	0.548	−0.047

Zarit = Zarit Burden Interview score; CUIDAR = Cuidar scale score; GHQ12 = General Health Questionnaire score; COPE = Spanish Version of the Brief COPE score; EPQRA = Personality Questionnaire Revised-Abbreviated score; Pfeiffer = Short portable mental status questionnaire score; Barthel = Barthel index score; df = degrees of freedom; *p* < 0.001 **.

**Table 3:** Prediction of burden in informal male caregivers.

Predictor	*β*	*t*	*p*	Lower 95% CI	Upper 95% CI	VIF
Time providing care (years)	0.337	3.872	<0.001 **	0.058	0.179	1.064
Caregiver’s competence (CUIDAR)	0.028	0335	0.739	−0.250	0.351	1.655
Caregiver’s social support (DUKE)	−0.050	−0.484	0.629	−0.415	0.252	1.457
Caregiver’s neuroticism (EPQRA)	0.349	3.798	<0.001 **	1.601	5.090	1.198
Elderly’s functional dependency (Barthel)	0.0416	4.592	<0.001 **	0.128	0.322	1.562
Elderly’s cognitive impairment (Pfeifer)	0.238	2.519	0.013 *	0.268	2.236	1.447

Zarit = Zarit Burden Interview score; CUIDAR= Cuidar scale score; GHQ12= General Health Questionnaire score; COPE = Spanish Version of the Brief COPE score; EPQRA = Personality Questionnaire Revised-Abbreviated score; Pfeiffer = Short portable mental status questionnaire score; Barthel = Barthel index score; *β* = Unstandardized Beta Coefficient; CI = Confidence Interval; VIF = Variance Inflation Factor; *p* < 0.05 *; *p* < 0.001 **.

**Table 4:** Prediction of burden in informal female caregivers.

Predictor	*β*	*t*	*p*	Lower 95% CI	Upper 95% CI	VIF
Time providing care (years)	0.258	5.992	<0.001 **	0.063	0.125	1.035
Caregiver’s competence (CUIDAR)	0.088	2.197	0.029	0.018	0.322	1.530
Caregiver’s social support (DUKE)	0.036	0.715	0.475	−0.102	0.218	1.442
Caregiver’s neuroticism (EPQRA)	0.528	11.218	<0.001 **	4.148	5.910	1.345
Elderly’s functional dependency (Barthel)	0.062	1.353	0.177	−0.015	0.082	1.285
Elderly’s cognitive impairment (Pfeifer)	0.230	4.938	<0.001 **	0.685	1.590	1.262

Zarit = Zarit Burden Interview score; CUIDAR = Cuidar scale score; GHQ12 = General Health Questionnaire score; COPE = Spanish Version of the Brief COPE score; EPQRA = Personality Questionnaire Revised-Abbreviated score; Pfeiffer = Short portable mental status questionnaire score; Barthel = Barthel index score; *β* = Unstandardized Beta Coefficient; CI = Confidence Interval; VIF = Variance Inflation Factor; *p* < 0.001 **.
